# From Xanthine Oxidase Inhibition to *In Vivo* Hypouricemic Effect: An Integrated Overview of *In Vitro* and *In Vivo* Studies with Focus on Natural Molecules and Analogues

**DOI:** 10.1155/2020/9531725

**Published:** 2020-02-25

**Authors:** João L. Serrano, Joana Figueiredo, Paulo Almeida, Samuel Silvestre

**Affiliations:** ^1^CICS-UBI, Health Sciences Research Center, University of Beira Interior, Av. Infante D. Henrique, 6200-506 Covilhã, Portugal; ^2^Department of Chemistry, University of Beira Interior, Rua Marquês de Ávila e Bolama, 6201-001 Covilhã, Portugal; ^3^CNC, Center for Neuroscience and Cell Biology, University of Coimbra, Rua Larga, 3004-517 Coimbra, Portugal

## Abstract

Hyperuricemia is characterized by elevated uric acid (UA) levels on blood, which can lead to gout, a common pathology. These high UA levels are associated with increased purine ingestion and metabolization and/or its decreased excretion. In this field, xanthine oxidase (XO), by converting hypoxanthine and xanthine to UA, plays an important role in hyperuricemia control. Based on limitations and adverse effects associated with the use of allopurinol and febuxostat, the most known approved drugs with XO inhibitory effect, the search for new molecules with XO activity is growing. However, despite the high number of studies, it was found that the majority of tested products with relevant XO inhibition were left out, and no further pharmacological evaluation was performed. Thus, in the present review, available information published in the past six years concerning isolated molecules with *in vitro* XO inhibition complemented with cytotoxicity evaluation as well as other relevant studies, including *in vivo* hypouricemic effect, and pharmacokinetic/pharmacodynamic profile was compiled. Interestingly, the analysis of data collected demonstrated that molecules from natural sources or their mimetics and semisynthetic derivatives constitute the majority of compounds being explored at the moment by means of *in vitro* and *in vivo* animal studies. Therefore, several of these molecules can be useful as lead compounds and some of them can even have the potential to be considered in the future clinical candidates for the treatment of hyperuricemia.

## 1. Introduction

Xanthine oxidase (XO) is a key enzyme in purine catabolism and, physiologically, catalyzes the oxidation of hypoxanthine to xanthine and then to uric acid (UA) with concomitant reactive oxygen species (ROS) production [1, 2]. Nonetheless, enzymes such as hypoxanthine-guanine phosphoribosyl transferase (HGPRT), phosphoribosyl pyrophosphate synthetase (PRPS), and phosphoribosyl pyrophosphate aminotransferase (PRPPAT) also participate in purine metabolic pathway [3]. The overproduction or low excretion of urate can lead to hyperuricemia and subsequently to gout [3, 4]. In the field of low excretion, anion-exchanging uptake transporters (OAT1, OCT2, URAT1, and GLUT9) have also been reported to play important roles in the regulation of serum UA [3].

**Figure 1:**
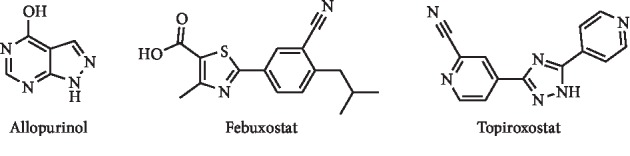
Structures of the clinically used XO inhibitors.

The most relevant target conditioning serum UA levels is XO, and excessive activity of this enzyme can lead to a pathological UA overproduction [5]. Therefore, its inhibition is of high interest, mainly in the treatment of gout, being allopurinol, febuxostat, and topiroxostat (Figure 1), the most known XO inhibitors. In addition to these clinically used drugs, over the years, several other molecules with XO inhibitory activity have been described [6–9]. Despite the existence of some reviews in this topic [6, 7, 9–11], it is necessary to complement these works with a study focusing on the *hit to lead* evolution in the development of new XO inhibitors with improved potency and safety when compared with the clinically used drugs. Interestingly, as can be seen in this review, natural molecules and semisynthetic analogues and derivatives constitute a large group of compounds being explored at the moment in this context.

## 2. Materials and Methods

The present review covers the literature published in the last 6 years and develops the most relevant studies that focus not only in XO inhibition but also in the integration of these data with other relevant information for the *hit to lead* evolution in the development of new XO inhibitors. In this view, cytotoxicity studies, *in vivo* hypouricemic effect and/or pharmacokinetic/pharmacodynamic profile, are also discussed. For this, a research on scientific databases Web of Science, Pubmed, Scopus, and others was carried out using associations of the following key terms and synonyms: XO, cytotoxicity, hyperuricemia, hypouricemic, or pharmacokinetic profile.

The review is organized according to the origin of the compounds (synthetic and natural/semisynthetics/mimetics of natural structures) and then according to their chemical structures. The structures of the scaffolds and/or the specific compounds with the most promising results are presented in figures.

## 3. Results

### 3.1. Synthetic Derivatives

#### 3.1.1. Purine Nucleus Analogues

**Figure 2:**
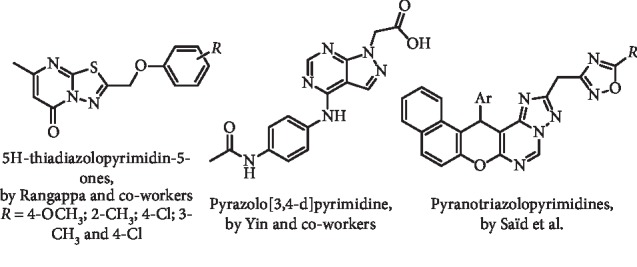
Structures of analogues of the purine nucleus.

Based on allopurinol structure, Rangappa and coworkers reported four 5H-thiadiazolopyrimidin-5-one analogues (Figure 2) as potent XO inhibitors with IC_50_ values in the range from 269 to 634 nM (XO from bovine milk) [12]. These compounds were later investigated on oxonate-treated rats, and it was observed that serum UA and creatinine levels significantly dropped at doses of 50 and 100 mg/kg. On the other hand, these pyrimidine analogues could significantly attenuate liver and kidney necrosis in oxonate-treated rats [13].

A series of pyrazolo[4,3-d]pyrimidine analogues (Figure 2) was prepared and evaluated by Yin and coworkers as XO inhibitors and was determined an IC_50_ value of 33.77 *μ*g/mL for the most potent inhibitor. Furthermore, most of these pyrimidines presented IC_50_ values higher than 64 *μ*g/mL against two human hepatocellular carcinoma cells [14]. More recently, this research group described novel purine derivatives with antitumoral effects and attenuated *in vitro* XO inhibition [15].

Saïd et al. performed an *in vitro* screening of novel pyranotriazolopyrimidines (Figure 2) at 100 *μ*M for the inhibition of XO activity and determined their cytotoxic effect against four cancer cell lines. According to the results, the XO% of inhibition at 100 *μ*M ranged between 4.4 and 25.5%, and relevant antiproliferative effects were observed [16].

#### 3.1.2. Febuxostat Analogues

**Figure 3:**
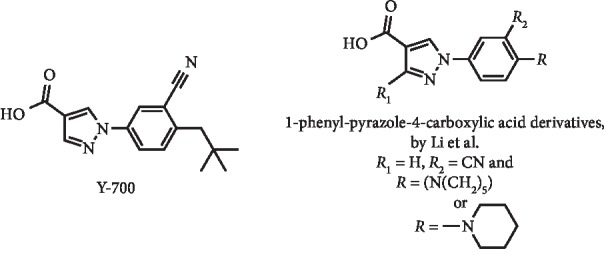
Structures of pyrazole febuxostat analogues.

Based on the success of the drug febuxostat, two series of 1-phenyl-pyrazole-4-carboxylic acid derivatives (Figure 3) have been designed and synthesized by Li et al. They reported a high *in vitro* XO inhibition, with IC_50_ values in the nanomolar range, acting by a mixed-type inhibition mechanism. The most potent inhibitors (IC_50_ of 4.2 and 5.7 nM) were further *in vivo* tested in mice with induced acute hyperuricemia, in comparison with the standard inhibitors febuxostat and Y-700 (Figure 3). Both studied compounds decreased the UA levels from approximately 1000 to 200 *μ*M after 5 hours at the dose of 5 mg/kg. Interestingly, the compound with IC_50_ = 5.7 nM (Figure 3, *R* = 1-piperidin-1-yl) presented the most promising hypouricemic effect, which was similar to that observed with febuxostat. However, the pharmacokinetic studies demonstrated that further strategy to improve the plasma concentration of this compound is required because the *C*_max_ and AUC_0−∞_ were remarkably lower than that determined for Y-700 [17].

**Figure 4:**
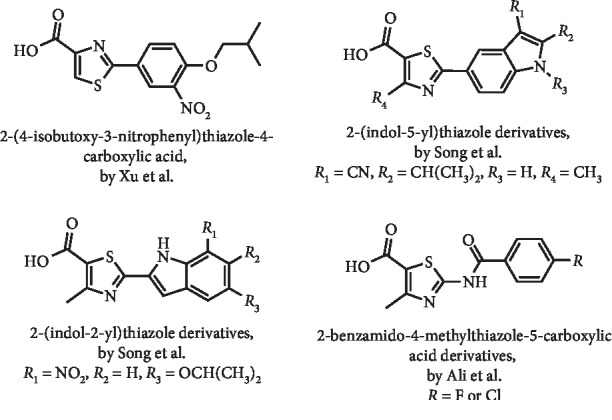
Structures of thiazole febuxostat analogues.

In a study aiming to develop other febuxostat analogues with improved pharmacological properties, Xu et al. prepared a series of compounds structurally similar to this drug mainly exploring different 5-membered heterocyclic rings. Considering these data from *in vitro* XO inhibition, it was demonstrated that 2-phenylthiazole-4-carboxylic acid can be considered a new scaffold for this activity. Of the compounds studied, the best result was observed with 2-(4-isobutoxy-3-nitrophenyl)thiazole-4-carboxylic acid (IC_50_ = 48.6 *μ*M; IC_50_ for febuxostat = 4.8 *μ*M). Interestingly, a hypouricemic effect of this compound (Figure 4) in potassium oxonate hypoxanthine-induced hyperuricemic mice was observed, however, inferior to that observed with febuxostat [18].

Also considering the febuxostat structure, Song et al. described a series of thiazole derivatives bearing the 2-(indol-5-yl) or 2-(indol-2-yl) moiety (Figure 4) [19, 20]. In the first study, they explored the structure-activity relationship for 2-(indol-5-yl)thiazoles, which presented XO inhibition levels similar to febuxostat, with IC_50_ values between 3.0 and 16 nM in most of the cases. However, not all of these compounds have this activity confirmed in an oxonic acid–induced high-uric acid *in vivo* model. In this context, 2-(3-cyano-2-isopropylindol-5-yl)-4-methylthiazole-5-carboxylic acid (Figure 4) exhibits the best compromise between *in vitro* XO inhibitory activity (IC_50_ of 3.5 nM) and plasma UA-lowering activity (60% at 1 h and 10 mg/kg). The pharmacokinetics profile study of this compound showed excellent oral bioavailability and long half-life. Therefore, according to the authors, these results evidenced the interest in performing clinical studies with this compound [19]. More recently, the same research group presented structure-activity relationship data for analogous 2-(indol-2-yl)thiazoles (Figure 4). From the observed results, although not being the most promising *in vitro* (IC_50_ of 5.1 nM) compound, 2-(7-nitro-5-isopropoxy-indol-2-yl)-4-methylthiazole-5-carboxylic acid (Figure 4) exhibited the most potent UA-lowering activity in the potassium oxonate–induced hyperuricemic rat model (43% at 1 h and 10 mg/kg). Similarly to which was observed with the previously referred 2-(indol-5-yl)thiazole derivative, this 2-(indol-2-yl)thiazole has excellent oral bioavailability in pharmacokinetic studies [20].

Ali et al. synthesized a different series of substituted 2-benzamido-4-methylthiazole-5-carboxylic acid derivatives (Figure 4) as potential XO inhibitors and free radical scavengers. *In vitro* studies revealed that the presence of a fluoro or chloro group at the *para* position of the benzamide led to an excellent XO inhibitory activity, with IC_50_ values of 0.57 (in a mixed-type inhibition) and 0.91 *μ*M, respectively. These results were confirmed in a potassium oxonate–induced hyperuricemic *in vivo* rat model, with 62 and 53% of UA inhibition being observed after 1 h at a dose of 10 mg/kg [21].

**Figure 5:**
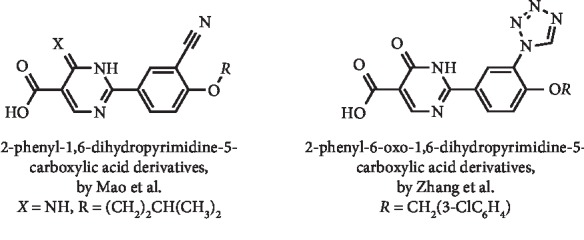
Structures of dihydropyrimidine febuxostat analogues.

Mao et al. reported a series of 2-phenyl-1,6-dihydropyrimidine-5-carboxylic acid derivatives (Figure 5) as excellent XO inhibitors with calculated IC_50_ values between 0.0181 and 0.5677 *μ*M. These IC_50_ values were substantially better than that reported for allopurinol (IC_50_ = 7.5902 *μ*M) and comparable to that of febuxostat (IC_50_ = 0.0236 *μ*M). The authors chose 2-(3-cyano-4-isopentyloxyphenyl)-6-imino-1,6-dihydropyrimidine-5-carboxylic acid (Figure 5), with an IC_50_ value of 0.0240 *μ*M, as a representative compound to continue the study. This derivative act as a mixed-type inhibitor, with a *K*_*i*_ value of 0.0042 *μ*M. Also, this compound was able to significantly reduce the serum concentration of UA at a single oral dose of 5 mg/kg on potassium oxonate-induced hyperuricemic mice, with a hypouricemic potency similar to allopurinol (dose = 10 mg/kg). In an acute oral toxicity study, this derivative did not lead to behavioral abnormality 24 h after the administration of a single dose of 2000 mg/kg, which suggested that the LD_50_ value might be higher than 2000 mg/kg, being near 400 times over the effective dose (5 mg/kg) [22].

Zhang et al. designed and prepared a group of 2-phenyl-6-oxo-1,6-dihydropyrimidine-5-carboxylic acid derivatives (Figure 5) bearing a tetrazol-1-yl and alkoxyl groups bounded to positions 3 and 4 of the phenyl ring, respectively, aiming to develop febuxostat analogues with higher selectivity for XO. Interestingly, all compounds had *in vitro* XO inhibitory properties (IC_50_ values ranging from 28.8 to 629 nM), and the most potent of these, 2-{4-[(3-chlorobenzyl)oxy]-3-(1H-tetrazol-1-yl)phenyl}-6-oxo-1,6-dihydropyrimidine-5-carboxylic acid (Figure 5), had an activity comparable to that observed with febuxostat (IC_50_ = 23.6 nM) and acted as a mixed-type inhibitor. The *in vivo* hypouricemic activity evaluation in the potassium oxonate–induced hyperuricemic rat model revealed that this compound (at an oral dose of 5 mg/kg) effectively reduced serum UA levels. However, its hypouricemic action was slightly lower than using febuxostat (5 mg/kg) and allopurinol (10 mg/kg). Furthermore, an acute oral toxicity study revealed that this dihydropyrimidine-5-carboxylic acid derivative was nontoxic to mice and could be tolerated at a dose up to 2000 mg/kg [23].

#### 3.1.3. Dihydropyrimidinone Derivatives

**Figure 6:**
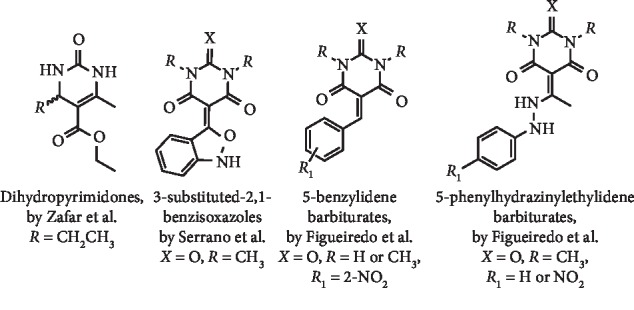
Structures of dihydropyrimidinones with XO inhibitory activity.

Taking into account their structural resemblance with the pyrimidine ring of xanthine, a series of dihydropyrimidinones (Figure 6) were prepared and evaluated by Zafar et al. as potential XO inhibitors. Interestingly, from a set of 25 derivatives, 22 were found to be good to weak XO inhibitors (IC_50_ values ranging from 14.4 to 418 *μ*M). These compounds were found to act by a competitive, noncompetitive, or mixed manner for XO inhibition. The best IC_50_ value was described for ethyl 4-ethyl-6-methyl-2-oxo-1,2,3,4-tetrahydropyrimidine-5-carboxylate (Figure 6), being a noncompetitive inhibitor, with a *K*_*i*_ = 20.5 *μ*M. Additionally, no cytotoxic effects on mouse fibroblasts 3T3 at the concentration of 100 *μ*M were observed for the most promising dihydropyrimidinones [24].

Also bearing the pyrimidine scaffold in their structure, several barbiturate and thiobarbiturate derivatives (Figure 6) were described as moderate XO inhibitors [25–27]. The XO inhibition of 3-substituted-2,1-benzisoxazoles (Figure 6) was demonstrated by Serrano et al. with an IC_50_ value of 22.10 *μ*M being determined for the best compound. In addition, a marked reduction in proliferation at a 30 *μ*M concentration induced by this barbiturate in the MCF-7 cell line was observed [25]. Moreover, 5-benzylidene [26] and phenylhydrazinylethylidene [26, 27] 1,3-disubstituted barbiturates (Figure 6) were described by Figueiredo et al. as moderate XO inhibitors, with IC_50_ values between 24.3 and 31.5 *μ*M [26, 27]. The authors also demonstrated that these barbiturates have low cytotoxicity in normal human dermal fibroblasts. In fact, in a screening at 30 *μ*M, a relative cellular proliferation ranging from 65 to 79% was observed, and an IC_50_ value of 82.02 *μ*M was determined for the most cytotoxic barbiturate [26, 27].

#### 3.1.4. Other Organic Synthetic Derivatives

**Figure 7:**
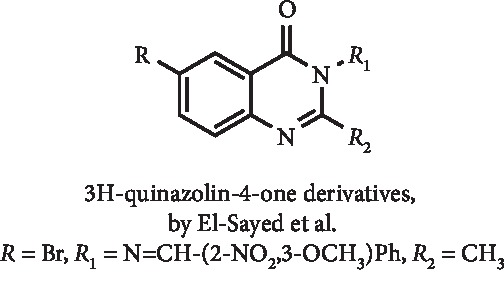
Structure of a 3H-quinazolin-4-one derivatives with XO inhibitory activity.

3H-Quinazolin-4-one derivatives (Figure 7) were synthesized by El-Sayed et al., and *in vitro* studies for XO inhibition demonstrated that none of these compounds evidenced better activity than allopurinol. For the best compound (Figure 7), an IC_50_ of 3.0 *μ*g/mL was calculated, which is a higher value than that observed for allopurinol (0.6 *μ*g/mL). Cytotoxicity studies on colorectal cancer HT-29 and SW620 cell lines showed cellular viability nearly 40% at the concentration of 30 *μ*g/mL [28].

**Figure 8:**
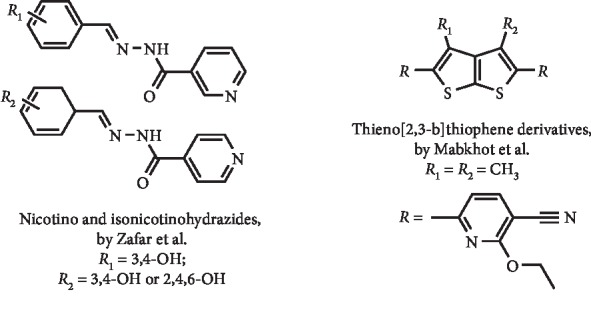
Structures of pyridine synthetic derivatives with XO inhibitory activity.

Benzylidene nicotino/isonicotinohydrazide derivatives (Figure 8) were reported by Zafar et al. as being good *in vitro* XO inhibitors, with IC_50_ values of 0.96, 10.0, and 12.4 *μ*M being determined for the three most potent compounds. In addition, it was demonstrated that these hydrazides act by a competitive mode of inhibition. These compounds were also found to be noncytotoxic against a mouse fibroblast cell line and thus were selected for *in vivo* studies. Interestingly, these authors demonstrated that two isonicotinohydrazides (Figure 8) were able to *in vivo* inhibit XO by 28 and 44% (at 50 mg/kg) against 100% observed for allopurinol at the same concentration [29].

A series of compounds incorporating the thieno[2,3-b]thiophene moiety (Figure 8) were synthesized and tested against several enzymes by Mabkhot et al. Three of these compounds demonstrated to be selective XO inhibitors, being determined for the most potent one (Figure 8) an IC_50_ value of 14.4 *μ*M. This compound was also found to be noncytotoxic in a human prostate cancer cell line [30].

**Figure 9:**
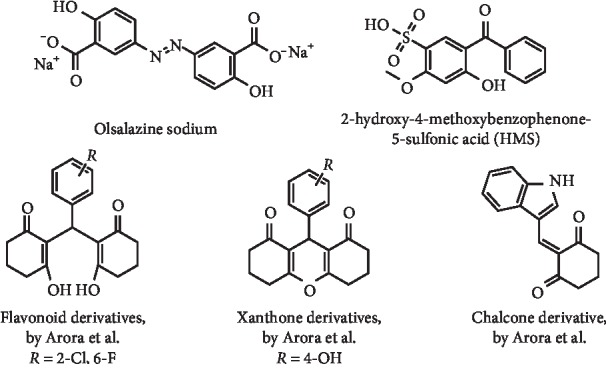
Structures of olsalazine sodium, 2-hydroxy-4-methoxybenzophenone-5-sulfonic acid, and Knoevenagel/tandem Knoevenagel and Michael adducts of cyclohexane-1,3-dione and aryl aldehydes.

Under the paradigm “old drug, new indication,” Niu et al. described olsalazine sodium (Figure 9), a commercial anti-ulcerative-colitis drug, as a serum UA levels reducer. For this, the author performed molecular docking to virtually screen potential XO inhibitors from a small approved drugs library. The *in vitro* studies showed olsalazine sodium as a promising compound in reducing XO activity, with an IC_50_ value of 3.4 mg/L (approximately 9.5 *μ*M), acting by a hybrid-type inhibition mode. Finally, it was observed in *in vivo* studies that this drug decreased serum UA levels and serum/hepatic XO activities after intraperitoneal administration. Nevertheless, as the onset of hypouricemic action for olsalazine occurred 4 h after administration, the authors suggested that this drug might be particularly suitable for gout prevention and long-term treatment [31].

2-Hydroxy-4-methoxybenzophenone-5-sulfonic acid (HMS) (Figure 9), a popular ultraviolet filter used in sunscreens, was studied as a potential hypouricemic agent by Zuo and coworkers. After an *in silico* molecular docking study, the XO inhibition by HMS was partially confirmed *in vitro*, and an IC_50_ of 36.1 *μ*M was obtained, in comparison with 11.4 *μ*M determined for allopurinol. In *in vivo* studies, it was observed that a 20 mg/kg dose of HMS administered to hyperuricemic mice leads to a significant reduction of serum UA levels. Possibly, this result was not only due to XO inhibition but also due to an upregulation of OAT1 and downregulation of GLUT9 mRNA and protein expression. Additionally, toxicity studies showed no negative impact in mice body weight growth and in kidney function [32].

Arora et al. designed and prepared Knoevenagel/tandem Knoevenagel and Michael adducts of cyclohexane-1,3-dione and aryl aldehydes (Figure 9) as XO inhibitors with a slight structural similarity to flavonoid (IC_50_ range from 4.93 to 15.28 *μ*M), xanthone (IC_50_ range from 4.08 to 13.14 *μ*M), and chalcone (IC_50_ = 3.66 *μ*M) scaffolds. Most of the described compounds presented better IC_50_ values than allopurinol (IC_50_ = 7.08 *μ*M). 2,2-((2-chloro-6-fluorophenyl)methylene)-bis(3-hydroxycyclohex-2-en-1-one) and 2-((1H-indol-3-yl)methylene)cyclohexane-1,3-dione (Figure 9) acted as noncompetitive and competitive-type inhibitors, respectively. Additionally, no significant cytotoxicity at 10 *μ*M on normal cells (HEK293) was observed for these compounds [33].

#### 3.1.5. Metal Complexes

**Figure 10:**
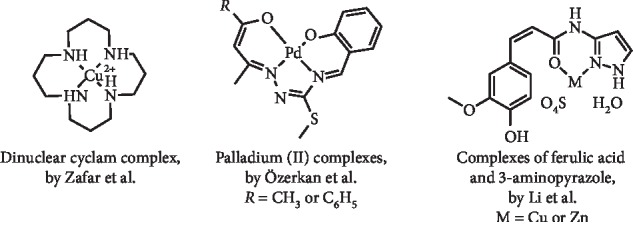
Structures of metal complexes with XO inhibitory activity and decreasing effect of serum UA levels.

A dinuclear cyclam complex (Figure 10) was described as a XO inhibitor (IC_50_ = 3.70 *μ*M) by Zafar et al. This complex acted by a noncompetitive type of inhibition and was also found to be *in vitro* noncytotoxic and led to a marked inhibition of *in vivo* XO activity [34]. Özerkan et al. reported the synthesis and evaluation of novel palladium (II) complexes with tetradentate thiosemicarbazones (Figure 10). Two compounds are interesting XO inhibitors (uncompetitive inhibitory mode), with IC_50_ of 0.4 and 0.7 *μ*g/mL, better values than the control allopurinol (IC_50_ = 1.1 *μ*g/mL). In addition, *in vitro* cytotoxicity studies on healthy 3T3 fibroblast cells demonstrated IC_50_ values of 7.1 and 6.2 *μ*g/mL for these two compounds, respectively [35].

Complexes resulting from the combination of ferulic acid, a known XO inhibitor, and 3-aminopyrazole, a molecule with anti-inflammatory effects, and copper or zinc (Figure 10) were prepared by Li et al. These two compounds exhibited inhibitory effects of XO activity in mouse liver homogenates, with better results being observed for the copper complex. In addition, studies in hyperuricemia model mice revealed that the elevated levels of blood UA could be decreased by both complexes (10 mg/kg, intraperitoneally injected) and that their effect is similar to the observed with febuxostat. Furthermore, it was evidenced that these complexes had no relevant effect on serum creatinine values, indicative of potential low renal side effects [36].

### 3.2. Natural and Semisynthetic Compounds and Mimetics of Natural Structures

#### 3.2.1. Phenolic Compounds and Analogues

**Figure 11:**
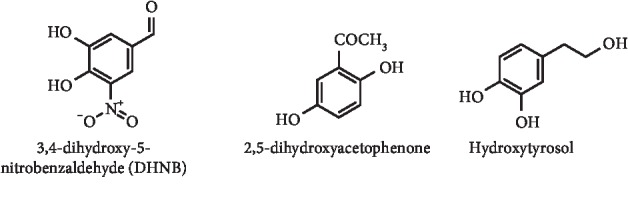
Structures of simple phenolic compounds with XO inhibitory activity and decreasing effect of serum UA levels.

*(1) Simple Phenolic Compounds*. Aiming to develop a novel XO inhibitor with a potent activity and low toxicity, Lü et al. described a series of natural catechols and analogues. Of these, 3,4-dihydroxy-5-nitrobenzaldehyde (DHNB) (Figure 11), structurally similar to protocatechuic aldehyde, demonstrated to be the most potent XO inhibitor, with an IC_50_ value of 3 *μ*M, acting by a mixed-type inhibition. In addition, DHNB effectively reduced serum UA levels after oral administration in allantoxanamide-induced hyperuricemic mice at a dose of 100 mg/kg. Nevertheless, *in vivo* toxicity studies demonstrated that a large oral dose of 500 mg/kg of DHNB did not lead to any evident side effects, in contrast with 42% of death in mice treated with the same dose of allopurinol [37]. The hypouricemic effect of 2,5-dihydroxyacetophenone (Figure 11), a compound computationally screened from *Ganoderma applanatum* was explored by Liang et al. For this, the *in vitro* and *in vivo* XO inhibitory activity was studied, evidence that this compound is an inhibitor of this enzyme (*in vitro* IC_50_ = 8.12 *μ*M). In hyperuricemic mice, it was also demonstrated that after oral administration of this phenolic compound (20, 40, and 80 mg/kg) serum UA was markedly reduced and that blood nitrogen and creatinine levels were lower than that observed in hyperuricemic and allopurinol controls. By means of RT-PCR and Western blot, further studies on the mechanism of action were performed, and it was verified that RNA and protein expressions of OAT1 (organic anion transporter 1) were upregulated and that the expressions of GLUT9 (glucose transporter 9), URAT1 (uric acid transporter 1), and CNT2 (gastrointestinal concentrative nucleoside transporter 2) were downregulated [38].

Hydroxytyrosol (Figure 11) is another natural catecholic compound that was studied by Wan et al. against XO by *in vivo* animal model and *in vitro* inhibition assay. Interestingly, hydroxytyrosol has XO inhibitory activity, with a determined IC_50_ value of 8.75 mM. This molecule can also decrease serum UA levels and adjust the mRNA transcription levels of UA transporter genes. In fact, this compound reduced reabsorption transporter genes mRNA and increased secretion transporter genes mRNA to the normal [39].

**Figure 12:**
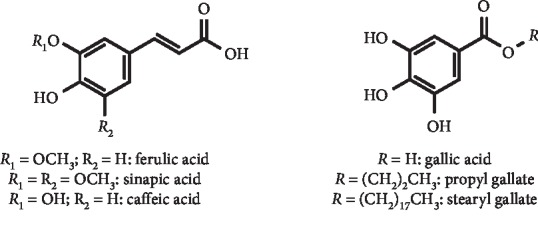
Structures of phenolic acids with XO inhibitory activity and decreasing effect of serum UA levels.

*(2) Phenolic Acids and Derivatives, Including Salvianolic Acids*. Several phenolic acids and derivatives were studied as XO and cyclooxygenase-2 inhibitors by Nile et al. According to these authors, all compounds showed good inhibition of XO activity. The most interesting IC_50_ values were found for sinapic acid and propyl and stearyl gallate (Figure 12). In addition, generally, they did not show significant *in vitro* cytotoxicity at 10 *μ*M except stearyl gallate; however, many of these ferulic acid derivatives revealed relevant toxicity at higher concentrations (50 *μ*M). Furthermore, interesting anti-inflammatory action was also observed for some of these compounds [40]. Caffeic acid (Figure 12), a known antioxidant belonging to the family of cinnamic acids and being present in numerous plants, also has XO inhibitory properties. Recently, Wan et al. evaluated its reducing effects on plasmatic UA levels in hyperuricemia rats and explored potential mechanisms of action in this context. Interestingly, it was demonstrated that serum UA levels were reduced after intragastric administration of caffeic acid (100 mg/kg) to hyperuricemia rats. In addition, blood urea nitrogen and serum creatinine levels decreased as well as *in vivo* XO and adenosine deaminase activities. Moreover, *in vitro* assays confirmed the XO inhibitory properties of this phenolic acid (IC_50_ = 53.45 *μ*M) and evidenced that it is a competitive inhibitor of this enzyme, which suffers structural changes (e.g. reduction of *α*-helix content) in the presence of this compound. Furthermore, it also acts by regulating the mRNA transcription of the renal uric acid transporters. In fact, the transcription levels of URAT1 and GLUT9 mRNA significantly increased, and the transcription levels of OAT1, UAT, and ABCG2 mRNA were significantly reduced than that observed in the blank group [41].

**Figure 13:**
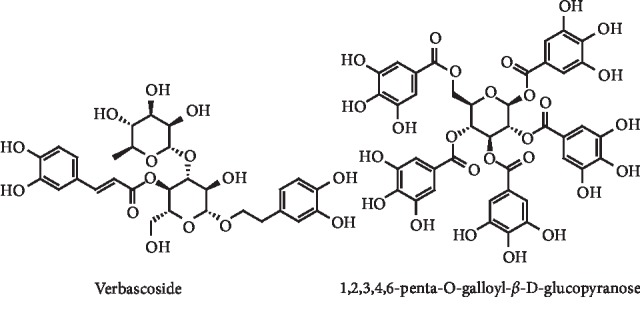
Structures of verbascoside and 1,2,3,4,6-penta-O-galloyl-*β*-D-glucopyranose.

Verbascoside, a cinnamate ester glycoside (Figure 13), also has *in vitro* inhibitory effect on XO (IC_50_ = 81.11 mg/mL). Additionally, a dose of 54 mg/kg of verbascoside in a hyperuricemic *in vivo* model could reduce serum UA to normal levels [42]. 1,2,3,4,6-Penta-O-galloyl-*β*-D-glucopyranose, another glycoside derivative, demonstrated *in vitro* inhibition of XO activity (IC_50_ = 2.8 *μ*M) in a noncompetitive manner (*K*_*i*_ = 3.1 *μ*M) with a potency closely to the observed with allopurinol (IC_50_ = 2.3 *μ*M). Serum UA levels on hyperuricemic mice at a dose of 40 mg/kg were also lowered [43].

**Figure 14:**
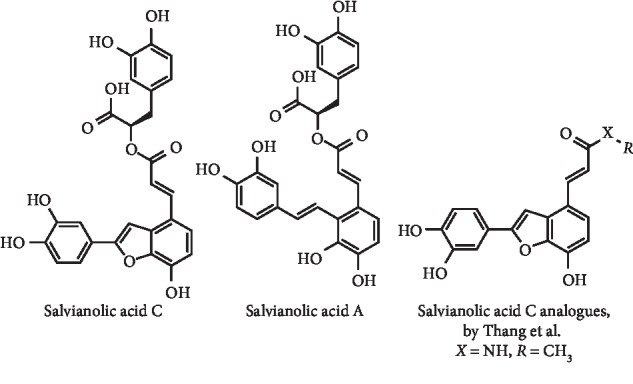
Structures of salvianolic acids C A and C analogues.

A combination of *in silico*, *in vitro*, and *in vivo* studies aiming to explore the interaction between ten representative chemicals identified in *Salvia miltiorrhiza* and XO were applied by Tang et al. Of these compounds, salvianolic acid C (Figure 14) proved to be the most potent *in vitro* XO inhibitor (IC_50_ = 8.79 *μ*M), acting by a mix-competitive manner. *In vivo* studies demonstrated a dose-dependent hypouricemic action of this compound in potassium oxonate–induced mice. Interestingly, it was observed that salvianolic acid C and allopurinol exhibit comparable *in vitro* XO inhibitory activity, but the hypouricemic effect of salvianolic acid C was lower [44]. Although with lower potency, salvianolic acid A (Figure 14) was also described as *in vitro* XO inhibitor, with an IC_50_ value of 73.17 *μ*M, and also led to a decrease UA levels in acute myocardial infarction rats [45, 46].

Thang et al. designed and synthesized a new series of 2-arylbenzo[b]furan derivatives (Figure 14), considering the structure and known bioactivity of salvianolic acid C. Most of these compounds exhibited potent *in vitro* XO inhibitory effect, particularly (E)-3-(2-(3,4-dihydroxyphenyl)-7-hydroxybenzofuran-4-yl)-N-methylacrylamide (IC_50_ = 4.45 *μ*M; IC_50_ of allopurinol = 10.61 *μ*M), which induced a mixed-type XO inhibition with a *K*_*i*_ of 3.5 *μ*M. Additionally, this compound (Figure 14) exhibit a dose-dependent hypouricemic action in potassium oxonate–induced hyperuricemic mice [47].

**Figure 15:**
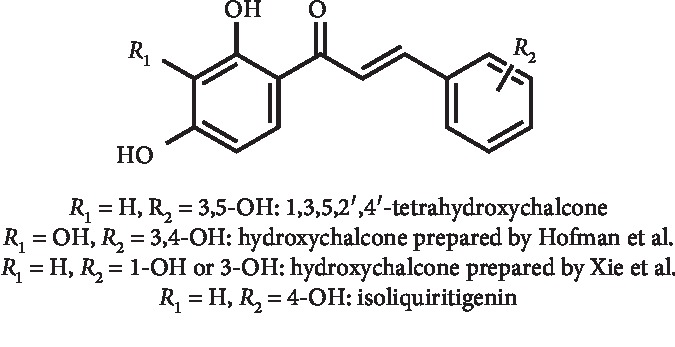
Structures of hydroxychalcones with XO inhibitory activity and decreasing effect of serum UA levels.

*(3) Hydroxylated Chalcones*, *6-Shogaol, Curcumin*, *and Analogues*. The chalcone scaffold, found in many naturally occurring compounds, have a wide range of bioactive properties and have also been prepared by synthesis [48, 49]. As an example, Li and co-workers [50–52] prepared several chalcones and evaluated their XO inhibitory activity. Of these, 3,5,2′,4′-tetrahydroxychalcone (Figure 15) displayed an interesting XO inhibition in a competitive manner, and IC_50_ and *K*_*i*_ values of 22.5 and 17.4 *μ*M, respectively, were determined [52]. In addition, *in vivo* intragastric administration of this chalcone significantly reduced serum UA levels in hyperuricemic mice in a dose-dependent manner. The best effect occurred with doses between 2 and 4 mg/kg [50–52]. Studies performed by the authors suggested that this chalcone act by a dual mechanism [50]. In fact, this *in vivo* hypouricemic action can be associated not only with the inhibition of key enzymes (XO, PRPS, PRPPAT, and HGPRT) in the purine metabolism but also with an enhancement in UA excretion by inhibiting the expression of GLUT9 in the kidney [50, 51]. Finally, an acute toxicity study in mice showed that 3,5,2′,4′-tetrahydroxychalcone was safe at a dose up to 5 g/kg, making it suitable for future studies [52]. Other hydroxylated chalcones prepared by a Claisen–Schmidt condensation were described by Hofmann et al., and IC_50_ values between 1.2 and 93 *μ*M as XO inhibitors were determined. Cytotoxicity tests revealed that the most active of these chalcones is noncytotoxic. However, further structure modifications on the chalcone scaffold are needed to optimize the XO inhibitory activity [53]. Later, Xie et al. described a synthetic series of hydroxychalcones (Figure 15) with moderate XO inhibition, and *in vitro* IC_50_ values of 47.3 and 56.8 *μ*M were calculated for the most potent ones. These hydroxychalcones, which act by a mixed XO inhibition type, were tested *in vivo* in potassium oxonate–induced hyperuricemic mice (10 and 50 mg/kg, intragastrically administered). The results demonstrated that they led to a significant serum UA and XO activity reduction in both cases, with the highest significance at 50 mg/kg. Acute toxicity *in vivo* studies revealed no evident toxicity at doses up to 5 g/kg [54].

The XO inhibition in a mixed manner by isoliquiritigenin (Figure 15) was initially described by Cheng and coworkers (IC_50_ and *K*_*i*_ values of 55.8 and 17.4 *μ*M, respectively) [55]. However, its poor absolute *in vivo* bioavailability was later reported [56]. In order to change that, Zang et al. developed an isoliquiritigenin-loaded self-microemulsifying drug delivery system. In fact, the *in vivo* oral bioavailability was enhanced in about 4.7 times, with an improvement in plasmatic concentrations. Additionally, the use of self-microemulsification proved to significantly improve the antihyperuricemic effect of isoliquiritigenin in rats. At the administered doses of 50–150 mg/kg, a reduction in serum UA levels from 25.2–51.9 to 41.7–78.8% for free drug and its drug delivery system was, respectively, demonstrated [57].

**Figure 16:**
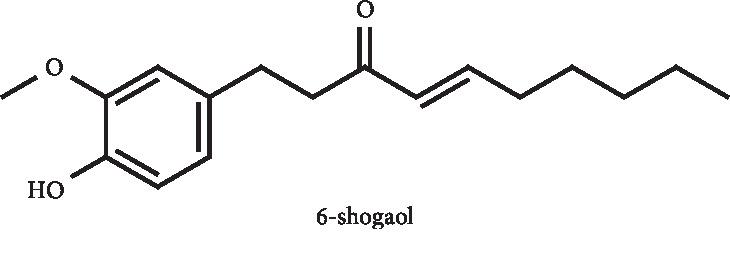
Structure of 6-shogaol.

A study conducted by Peng et al. demonstrated that phenolic compounds isolated from ginger inhibit the XO activity at the concentration of 125 *μ*M. 6-Shogaol (Figure 16), one of these phenols, also presented IC_50_ values of relative cell proliferation between 7.4 and 100.0 *μ*M in the different tested cancer cell lines [58]. Later, Nile and Park reported again the XO inhibition by 6-shogaol, with an IC_50_ value of 15.2 *μ*M *versus* 8.4 *μ*M of allopurinol [59]. Due to the poor water solubility of 6-shogaol, Yu, Xu, and coworkers developed 6-shogaol-loaded solid lipid nanoparticles [60] and a 6-shogaol-loaded self-microemulsifying drug delivery system [61] to enhance its oral bioavailability. In fact, the nanoencapsulation led to a significant improvement of *in vivo* oral absorption, bioavailability, and acting time of 6-shogaol in healthy rats. 6-Shogaol–loaded solid lipid nanoparticles also could lower the serum UA levels in a higher extension than free drug in hyperuricemia/gouty arthritis rats at a 120 mg/kg dose. In addition, loaded solid lipid nanoparticles allowed an improvement in the organ protection effects of 6-shogaol [60]. The use of a 6-shogaol–loaded self-microemulsifying drug delivery system led to an increment of 571.18% in the oral bioavailability when compared with the administration of the free drug. In addition, an increment in the reduction of serum UA levels in hyperuricemic rats treated with 25, 50, and 100 mg/kg of dose was observed. As example, the highest dose (10 mg/Kg) led to a reduction in UA levels by 71.1% by the microemulsification and 60.0% by free drug. The 6-shogaol–loaded self-microemulsifying drug delivery system was also pointed as promising to reduce the kidney damage caused by hyperuricemia model [61].

**Figure 17:**
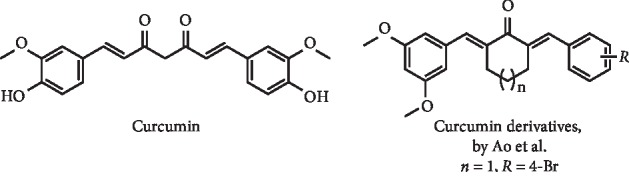
Structure of curcumin and analogues with XO inhibitory activity and decreasing effect of serum UA levels.

Curcumin (Figure 17) was described as a XO inhibitor, with IC_50_ of 117.3 *μ*M (IC_50_ of allopurinol = 28.9 *μ*M) by Peng et al. [58]. Later, Chen et al. reported its *in vivo* activity on potassium oxonate–induced hyperuricemia mice. Oral administration in a dose of 20 or 40 mg/kg leads to a decrease of serum UA and an effective inhibition of serum and liver XO levels and to a reduction of kidney inflammation by NLRP3 inflammasome suppression [62]. *α*,*β*-Unsaturated cyclohexanone and cyclopentanone analogues of curcumin (Figure 17) were described as potential antihyperuricemia agents, acting by dual inhibition of XO and URAT1 by Ao et al. The most promising compound (Figure 17) with *in vitro* dual inhibition also demonstrated to reduce serum UA levels in a hyperuricemic *in vivo* model. Interestingly, at the doses of 10 and 20 mg/kg, this curcumin derivative increased the urinary UA excretion and decreased the serum and hepatic UA levels as well as XO activity and URAT1 protein levels. According to these authors, this compound is a first-in-class of dual inhibitors and may serve as a reference compound for further design of antihyperuricemic drugs [63].

**Figure 18:**
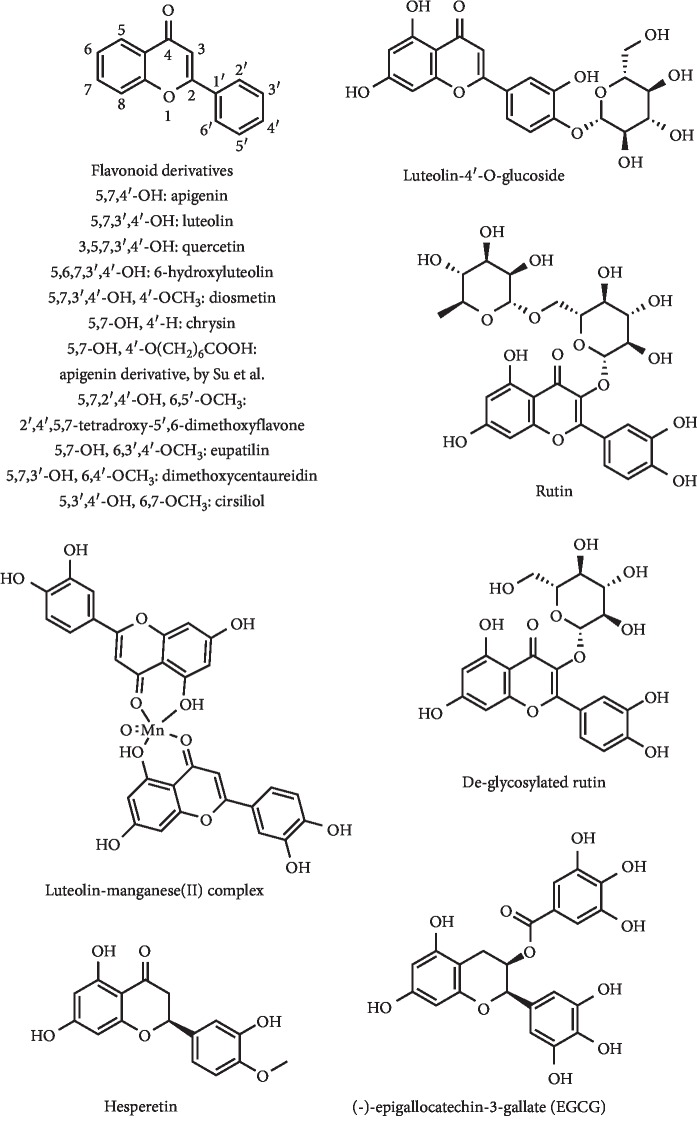
Structures of flavonoids with XO inhibitory activity and decreasing effect of serum UA levels.

*(4) Flavonoids and Analogues*. Natural plant flavonoids have always been molecules of great interest [64]. Their hypouricemic action in mice was also evidenced [65] as well as of some semisynthesized derivatives [66]. In fact, the flavonoids luteolin, apigenin, diosmetin, chrysin, O^3′^,O^7^-dihexyl diosmetin, O^4′^,O^7^-dihexyl apigenin, and O^7^-hexyl chrysin (Figure 18) showed XO inhibition, with IC_50_ values between 4.5 and 8.1 *μ*g/mL. In addition, anti-inflammatory action and a relatively low cytotoxicity were found for these flavonoids [66]. Other series of apigenin flavonoid derivatives have been prepared by Su et al. by a coupling of the carboxyl alkyl group to 4′-, 5- or 7-hydroxyl groups of apigenin. The 4′-modified derivatives (Figure 18) were demonstrated to be more potent than apigenin in XO inhibition, with IC_50_ values between 0.098 and 0.82 *μ*M, versus 3.2 *μ*M. The insertion of a carboxyl *n*-hexyl group in this position (Figure 18) led to the best *in vitro* results. This molecule was evaluated for its hypouricemic effects on the potassium oxonate–induced hyperuricemic mouse model. Interestingly, the serum UA levels were significantly decreased by an intraperitoneal dose of 10 mg/kg, with no significant effects being observed with apigenin. Nevertheless, the *in vitro* results presented higher XO inhibition for apigenin derivatives than allopurinol, but *in vivo* tests did not confirm these results. The authors pointed to the poor bioavailability or short half-life of the compound as the cause of this reduced activity [67].

Four flavones with XO inhibitory activity were described by Metoui et al. from the Tunisian *Artemisia campestris* leaves. 2′,4′,5,7-Tetrahydroxy-5′,6-dimethoxyflavone, eupatilin, dimethoxycentaureidin, and cirsiliol (Figure 18) exhibited higher potency for XO inhibition than allopurinol (IC_50_ = 8.2 *μ*M), with IC_50_ values of 5.5, 3.3, 6.8, and 5.5 *μ*M, respectively. In addition, these four flavones were *in vitro* tested against four cancer cell lines at a concentration of 15 *μ*M to verify their cytotoxic activity. A proliferation value below or close to 50% was found for all tested flavones at this single concentration [68].

Luteolin (Figure 18) has been reported to have a significant XO inhibitory activity in comparison with allopurinol, with an IC_50_ value of 4.8 *μ*M [69]. An inhibition effect assay performed by Dong et al. evidenced that luteolin and a luteolin–manganese(II) complex (Figure 18) reversibly inhibited XO in a competitive manner. Interestingly, the complex had a more remarkable hypouricemic effect than luteolin and both compounds proved to be noncytotoxic to a human liver cancer cell line [70]. A glycosylated derivative, luteolin-4′-glucoside (Figure 18), also inhibited XO, with an *in vitro* IC_50_ value of 0.26 *μ*g/mL [71]. In addition, this molecule had potent uricosuric effects in hyperuricemic mice with renal mURAT1 and decreasing XO activity, as well as anti-inflammatory effects. Furthermore, it was noted that, at the same concentration of luteolin and luteolin-4′-O-glucoside, a better therapeutic effect was observed for luteolin [72]. 6-Hydroxyluteolin (Figure 18) was also described as an inhibitor of XO activity, with an IC_50_ value of 7.52 *μ*M [73]. *In vivo* studies evidenced that this flavonol led to a significant dose-dependent reduction on the serum UA level of hyperuricemic rats at doses of 0.05, 0.1, and 0.3 mmol/kg. It was also demonstrated significant inhibition of rat liver XO (about 80%) in the highest dose [74].

de Araújo et al. described the improvement of *in vitro* XO inhibitory activity after enzymatic deglycosylation of rutin (Figure 18). However, this hydrolysis also leads to an increase in the cytotoxicity of this flavonoid against several tested cell lines. The total deglycosylated derivative, quercetin (Figure 18), was demonstrated to be the most potent XO inhibitor and had intermediary cytotoxicity [75]. In this context, it was previously demonstrated that quercetin has high XO inhibitory effects, with an IC_50_ value of 1.9 *μ*M [76]. Glycosylated anthocyanins from purple sweet potato [77–80] as well as other glycosylated flavonoids and coumaroylspermidines from rape bee pollen [81] were described by Zhang research group as good XO inhibitors. In fact, these authors demonstrated that these compounds, particularly highly acylated anthocyanins, can reduce the kidney inflammation in hyperuricemic mice in addition to their hypouricemic effect [77, 80].

The flavonoids hesperetin (Figure 18), hesperidin, and G-hesperidin were also tested as XO inhibitors by De Souza et al. These authors demonstrated that hesperetin has more potent *in vitro* XO inhibitory activity than the glycosylated derivatives (hesperidin and G-hesperidin). This compound acted by a competitive inhibition mode, and IC_50_ and *K*_*i*_ values of 53 and 17 *μ*M, respectively, were determined. In addition, *in vivo* studies demonstrated that monoglucuronides of hesperetin were the major forms present in plasma after the ingestion of this flavonoid [82].

(−)-Epigallocatechin-3-gallate (EGCG) (Figure 18), important catechin of green tea, was evaluated as XO inhibitor, with a determined IC_50_ of 12.5 *μ*M by Lin et al. These authors described that EGCG may act as “suicide substrate,” similarly to allopurinol [83]. More recently, Zhu et al. demonstrated that EGCG displayed inhibitory effects on hepatic XO at the dose of 50 mg/kg. In addition, this compound had a significant dose-dependent effect on lowering serum UA levels and in the regulation of GLUT9 and URAT1 mRNA expression levels in hyperuricemic mice, when compared with the model group. Furthermore, this molecule had effective renal protective effects in the prevention of glomeruli and kidney tubules damages, as evidenced by the analysis of histopathologic sections of hyperuricemic mice [84].

**Figure 19:**
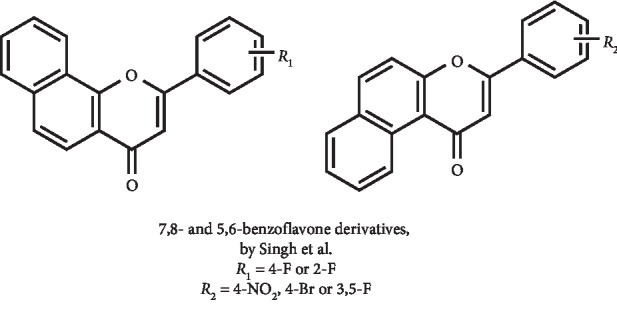
Benzoflavone derivatives with XO inhibitory activity and decreasing effect of serum UA levels.

**Figure 20:**
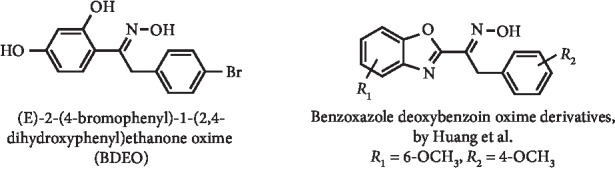
Structures of oxime analogues of flavonoids with XO inhibitory activity and decreasing effect of serum UA levels.

Two series of benzoflavone derivatives (Figure 19) were designed, synthesized, and evaluated for their XO inhibitory potential by Singh et al. Some of these derivatives had significant *in vitro* XO inhibition, with IC_50_ values lower than 10 *μ*M. Among the series, the most potent compounds on *in vitro* studies were 7,8- and 5,6-benzoflavones (Figure 19), which demonstrated IC_50_ values of 0.6 and 5.2 *μ*M, respectively. These derivatives act as mixed-type inhibitors and has a higher potency than allopurinol (IC_50_ = 8.7 *μ*M). Of these two compounds, *in vivo* studies demonstrated that only the 5,6-benzoflavone derivative presented the ability to reduce the serum UA to its normal level at a dose of 10 mg/kg. Interestingly, the authors explored six other derivatives on *in vivo* studies that evidenced the potential of at least three compounds as clinical candidates for the treatment of hyperuricemia. These compounds, which presented *in vitro* IC_50_ values of 4.9, 8.9, and 7.7 *μ*M, showed to be able to reduce *in vivo* serum UA to values at a level comparable to that observed with febuxostat (dose of 5 mg/kg). Additionally, two of these benzoflavones did not led to behavioral abnormality on *in vivo* acute toxicity study [85].

(E)-2-(4-Bromophenyl)-1-(2,4-dihydroxyphenyl)ethanone oxime (BDEO) (Figure 20), a novel compound with a chemical structure similar to flavonoids, was described as an inhibitor of XO activity, with an IC_50_ value of 3.33 *μ*M. Its UA uptake via URAT1 was evaluated *in vitro*, and the results demonstrated that this absorption was effectively inhibited. According to this study, BDEO effectively decreased hepatic XO activity and downregulated renal URAT1 protein expression in hyperuricemic mice, especially at doses of 10 and 20 mg/kg. Nevertheless, those indicators were not changed by BDEO in normal mice when compared with allopurinol and benzbromarone, reflecting its marked advantages in treating hyperuricemia and predicting its safety. Therefore, the authors suggested that BDEO may serve as a dual XO and URAT1 inhibitor for the treatment of hyperuricemia [86]. Considering the structure of flavonoids and synthetic intermediates for their preparation with antihyperuricemia effects, XO inhibition and also immune-regulating actions, several benzoxazole analogues of these compounds (Figure 20) were developed aiming to improve these activities. Specifically, two series of benzoxazole deoxybenzoin oxime derivatives were prepared as dual inhibitors of innate immune sensors and XO. *In vitro* studies evidenced that the majority of compounds suppressed XO activity, and the best result was observed with compound (E)-1-(6-methoxybenzo[d]oxazol-2-yl)-2-(4-methoxyphenyl)ethanone oxime (IC_50_ = 3.7 *μ*M; IC_50_ for allopurinol = 2.9 *μ*M). Enzyme kinetics studies demonstrated that this compound (Figure 20) acted as a competitive-type XO inhibitor. Using mice with oxonate-induced hyperuricemia, this compound reduced serum UA levels in a dose-dependent manner, consistent with *in vitro* studies. In addition, it was demonstrated that this benzoxazole has higher safety than allopurinol in normal mice and that it has antiacute gouty arthritis effect *in vivo* [87].

**Figure 21:**
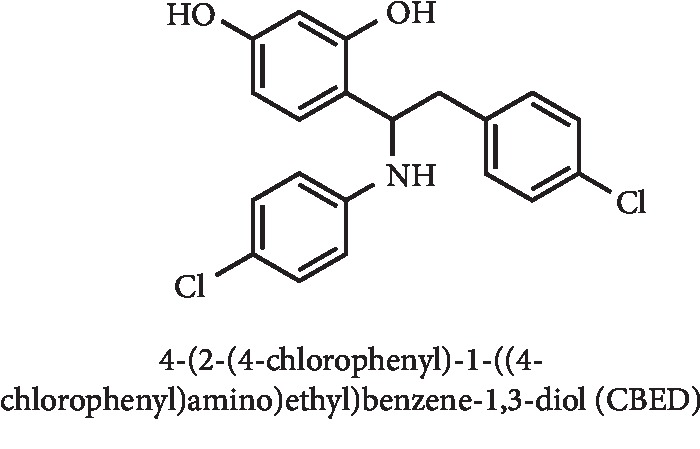
Structure of 4-(2-(4-chlorophenyl)-1-((4-chlorophenyl)amino)ethyl)benzene-1,3-diol (CBED).

From a set of compounds with a chemical structure similar to flavonoids, 4-(2-(4-chlorophenyl)-1-((4-chlorophenyl)amino)ethyl)benzene-1,3-diol (CBED) (Figure 21) was found to be a dual XO and NLRP3 inhibitor after molecular docking studies by Liu and coworkers. *In vitro* results for XO inhibitory activity demonstrated an IC_50_ value of 3.9 *μ*M, close to that obtained for allopurinol (IC_50_ = 2.1 *μ*M). On the other hand, *in vivo* results demonstrated a reduction in serum UA levels at the dose of 20 mg/kg. Additionally, a remarkably suppression of NLRP3 inflammasome activation and regulation on hepatic XO activity were observed. Thus and according to the authors, CBED can lead not only to a hypouricemic effect but also to a reduction in kidney inflammation caused by high UA levels [88].

**Figure 22:**
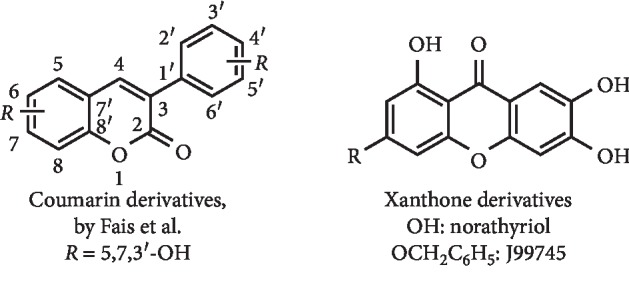
Structure of phenolic coumarin and of xanthone derivatives with XO inhibitory activity.

*(5) Phenolic Coumarin and Xanthone Derivatives*. To understand structure-activity relationships on a series of coumarin derivatives, Fais et al. prepared a series of twenty 3-arylcoumarins and eight 3-heteroarylcoumarins. Of these, 5,7-dihydroxy-3-(3′-hydroxyphenyl)coumarin (Figure 22) was proved to be the best XO inhibitor with a determined IC_50_ value 7-fold better than that observed for allopurinol (IC_50_ of 2.1 and 14.7 *μ*M, respectively). *In vitro* kinetics studies revealed an uncompetitive inhibition mode and a *K*_*i*_ value of 0.4 *μ*M. Finally, studies on 3T3 normal fibroblasts revealed no cytotoxic effect caused by this compound [89].

Norathyriol (Figure 22), a xanthone analogue, was described by Lin et al. as an uncompetitive concentration-dependent XO inhibitor, with IC_50_ of 7.8 *μ*M, close to that observed for allopurinol (IC_50_ = 76.3 *μ*M). The uncompetitive inhibition was appointed by the authors as a beneficial point in comparison with the competitive or mixed-type inhibition of allopurinol and febuxostat, respectively. In addition, an intragastric dose of 2.0 mg/kg of norathyriol was enough to reduce the serum UA levels in hyperuricemic mice to the normal values of healthy mice. *In vivo* results demonstrated also that norathyriol acted not only by XO inhibition but also by OAT1 activation [90]. Qin et al. demonstrated for the first time that the norathyriol 3-benzyloxy derivative J99745 (Figure 22) presented an *in vitro* XO inhibition, with a determined IC_50_ value of 3.297 *μ*M. Experiments involving hyperuricemia mice showed that J99745 at doses of 10 and 30 mg/kg significantly reduced serum urate levels, enhanced UA excretion, and provided higher nephroprotective effects than allopurinol. These authors suggested that this molecule exerts a urate-lowering effect by the inhibition of XO activity and a decrease in URAT1 expression [91].

#### 3.2.2. Terpenes and Dioscin

**Figure 23:**
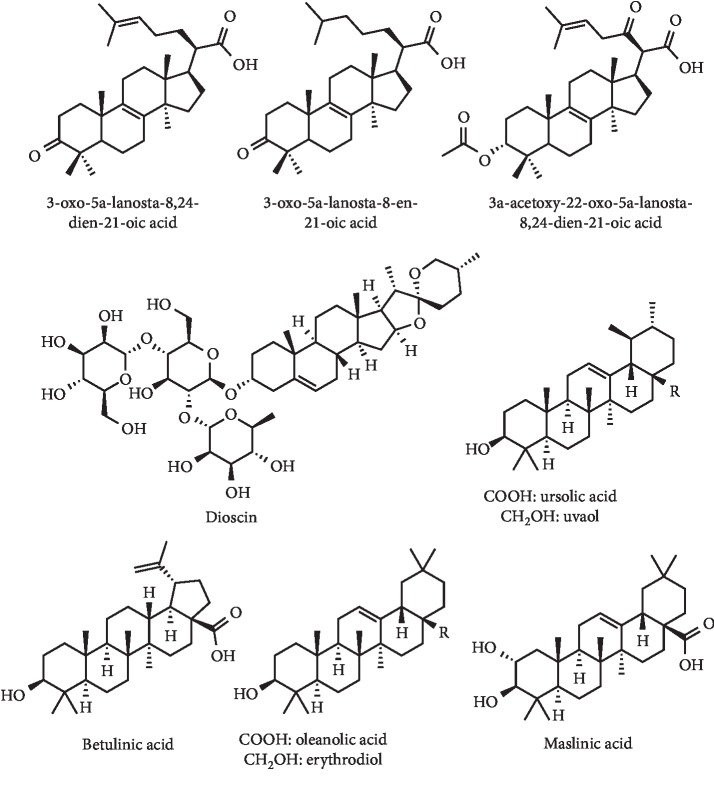
Terpenes and dioscin with XO inhibitory activity and decreasing effect of serum UA levels.

Lin et al. isolated a triterpenoid and an alkamide from *Ganoderma tsuga* and prepared four terpenoid derivatives by semisynthesis from 3-oxo-5*α*-lanosta-8,24-dien-21-oic acid (Figure 23). In addition, these compounds were evaluated as XO inhibitors, antioxidants, and antiproliferative agents against human prostatic cancer cells. Of these, two compounds with interesting XO inhibition (IC_50_ values of 313.3 and 43.9 *μ*M) in a concentration-dependent manner were identified. The compound with the best activity (3-oxo-5*α*-lanosta-8-en-21-oic acid) (Figure 23), prepared by semisynthesis, was as a mixed inhibitor of XO, with a *K*_*i*_ value of 3.2 *μ*M. The authors also reported the cytotoxicity of this compound against prostatic cancer cells, with an IC_50_ value of 23.9 *μ*M being determined [92]. In previous works of this research group, the lanostanoid used as starting material had already been isolated from *Ganoderma tsugae* [92, 93]. In addition, five another lanostanoids were isolated and tested as XO inhibitors and as DPPH scavengers, and their cytotoxicity was evaluated against prostatic cancer cells and keratinocytes. Of these, three compounds inhibited the XO activity in a concentration-dependent manner with IC_50_ values of 90.2, 116.1, and 181.8 *μ*M. The compound with the highest XO inhibition (3*α*-acetoxy-22-oxo-5*α*-lanosta-8,24-dien-21-oic acid) (Figure 23) is a mixed inhibitor with Ki of 0.6 *μ*M. However, this compound also presented the most interesting result in human prostatic cancer cells, also enhancing the cytotoxicity induced by cisplatin [93]. Since a long time, the glycoside dioscin (Figure 23) is known as a weak XO inhibitor with an IC_50_ value of 115 *μ*M [94]. Recently, it was evidenced that it has pronounced antihyperuricemic effects in mice by reducing serum UA levels over 4 h at 25 and 50 mg/kg of oral dose [95, 96]. This activity can be explained by the regulation of levels of mOAT1, mURAT1, mOCT2, and GLUT-9 expressions in the kidney [95, 96].

Ursolic acid (Figure 23), a bioactive pentacyclic triterpene extracted from the *Tribulus arabicus* hexane fraction, was found to be a potent XO inhibitor. The authors described an *in vitro* IC_50_ value of 10.3 *μ*g/mL for the inhibition of XO activity (IC_50_ = 6.5 *μ*g/mL for allopurinol). In addition, this *in vitro* activity was confirmed *in vivo* on mice with potassium oxonate–induced hyperuricemia. Results demonstrated an effective hypouricemic effect at a dose of 5 and 10 mg/kg with the reduction of UA levels in 56.1 and 79.9%, respectively [97]. Given its potential as a hypouricemic agent, ursolic acid is already patented in China for gout treatment [98]. In a recent study on triterpenic acids from apple pomace by Kai and coworkers, an *in vitro* cytotoxicity with IC_50_ values between 5.6 and 20.8 *μ*g/mL in four cancer cell lines was described for ursolic acid. From this natural source, other compounds were isolated and tested against XO. In fact, beyond the most active betulinic acid (IC_50_ = 12.6 *μ*g/mL), oleanolic acid (IC_50_ = 17.8 *μ*g/mL), maslinic acid (IC_50_ = 21.5 *μ*g/mL), erythrodiol (IC_50_ = 25.8 *μ*g/mL), and uvaol (IC_50_ = 30.2 *μ*g/mL) were also described (Figure 23). Nevertheless, all the described triterpenic acids did not show higher activity than allopurinol (IC_50_ = 9.6 *μ*g/mL). By means of kinetic studies, a noncompetitive type of inhibition for betulinic acid was revealed. Additionally, *in vitro* cytotoxic effects on four cancer cell lines and IC_50_ values from 6.2 to 40.1 *μ*g/mL were obtained [99].

#### 3.2.3. Other Natural and Semisynthetic Compounds

**Figure 24:**
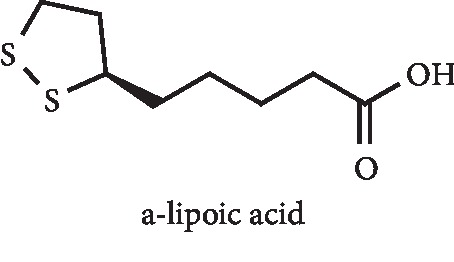
Structure of *α*-lipoic acid.

*α*-Lipoic acid (Figure 24) was found by Hameed and Ramadhan to be a potent XO inhibitor, with an IC_50_ value of 2.9 *μ*g/mL when compared with 1.7 *μ*g/mL of allopurinol. *In vivo* results on potassium oxonate–induced hyperuricemic mice demonstrated that a 10 mg/kg dose of *α*-lipoic acid and allopurinol led to a serum UA reduction from 4.4 mg/dL to 2.3 and 1.7 mg/dL, respectively [100].

**Figure 25:**
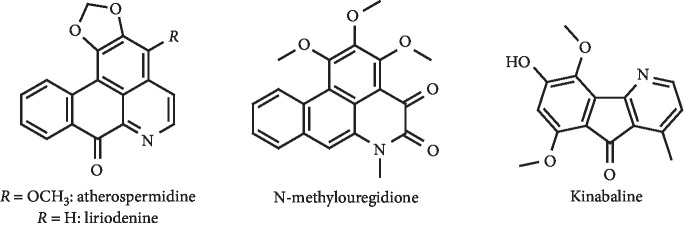
Structures of alkaloids with XO inhibitory activity.

The alkaloids from *Alphonsea elliptica* barks atherospermidine (IC_50_ = 46.3 *μ*g/mL), liriodenine (IC_50_ = 7.7 *μ*g/mL), *N*-methylouregidione (IC_50_ = 42.1 *μ*g/mL), and kinabaline (IC_50_ = 50.7 *μ*g/mL) (Figure 25) were described as possessing moderate inhibition of XO activity by Aldulaimi et al. In addition, the cytotoxicity on MCF-7 human breast cancer cells was evaluated, with determined IC_50_ values greater than 62.0 *μ*g/mL [101].

**Figure 26:**
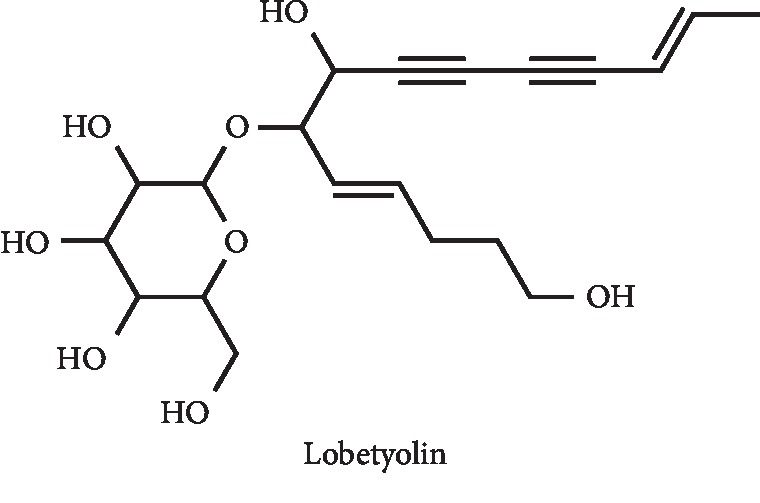
Structure of lobetyolin.

The effect of lobetyolin (Figure 26) on XO inhibition was presented by Yoon and Cho. Even though a weak *in vitro* mixed XO inhibition (IC_50_ = 2985 *μ*M), an *in vivo* significant hepatic XO activity reduction at a dose of 50 mg/kg was observed [102].

**Figure 27:**
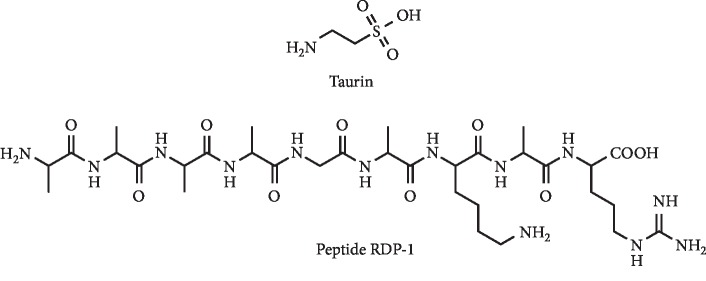
Structures of the amino acid taurine and of the peptide RDP-1.

In the field of small natural molecules, Feng et al. demonstrated that the administration of 1% taurine (Figure 27), a sulfur-containing semiessential amino acid, in drinking water could efficiently ameliorate kidney injury and decrease uric acid levels by regulating uric acid formation and excretion in hyperuricemic rats [103]. The significant inhibition of XO activity by taurine was already been evidenced [104].

A new peptide, RDP-1 (Figure 27), was isolated from the extract of shelled fruits of *Oryza sativa* and structurally characterized. *In vivo* biological evaluation evidenced that after intragastric administration, this compound (10 *μ*g/Kg) reduced hyperuricemia induced by potassium oxonate in rats. In addition, a reduction of creatinine levels and an ameliorating effect on hyperuricemic nephropathy was observed. It was also demonstrated that this peptide inhibited XO *in vitro* and *in vivo*, probably by occupying the binding site to xanthine (docking studies). Furthermore, no acute toxicity in rats was observed, and authors also evidenced that RDP-1 was stable in several temperatures [105].

## 4. Conclusions

Given the relevance of XO as a validated target to reduce increased serum UA levels and also due to the fact that the commercialized drugs with XO inhibitory effects can have serious side effects, in recent years a large number of new alternative XO inhibitors were described. Despite the fact that the majority of tested products with relevant *in vitro* XO inhibition do not advance for further pharmacological evaluation, it is clear in this review that a large number of compounds are being explored not only in *in vitro* studies but also in *in vivo* biological evaluation, and very promising molecules can be recognized and used in future studies. It is also possible to conclude that the majority of compounds being explored in more advanced biological evaluation studies are natural structures or their mimetics and semisynthetic derivatives. In addition, it is clear that frequently the observed *in vitro* XO inhibition results do not have a clear correspondence with the *in vivo* hypouricemic effects. This fact evidences the relevance of performing *in vivo* biological evaluation of the efficacy of the molecules in addition to the *in vitro* XO inhibition studies and access, as far as possible, other possible mechanisms of action, as well as the pharmacokinetics and toxicity profile of the molecules under study.
